# Rapid Prediction of Retina Stress and Strain Patterns in Soccer-Related Ocular Injury: Integrating Finite Element Analysis with Machine Learning Approach

**DOI:** 10.3390/diagnostics12071530

**Published:** 2022-06-23

**Authors:** Yasin Shokrollahi, Pengfei Dong, Mehmet Kaya, Donny W. Suh, Linxia Gu

**Affiliations:** 1 Department of Biomedical and Chemical Engineering and Sciences, Florida Institute of Technology, Melbourne, FL 32901, USA; yshokrollahi2020@my.fit.edu (Y.S.); pdong@fit.edu (P.D.); mkaya@fit.edu (M.K.); 2 Gavin Herbert Eye Institute (GHEI), University of California at Irvine, Irvine, CA 92697, USA; donnys@hs.uci.edu

**Keywords:** soccer-related ocular injuries, finite element analysis, retinal stress, retinal strain, machine learning, partial least squares regression

## Abstract

Soccer-related ocular injuries, especially retinal injuries, have attracted increasing attention. The mechanics of a flying soccer ball have induced abnormally higher retinal stresses and strains, and their correlation with retinal injuries has been characterized using the finite element (FE) method. However, FE simulations demand solid mechanical expertise and extensive computational time, both of which are difficult to adopt in clinical settings. This study proposes a framework that combines FE analysis with a machine learning (ML) approach for the fast prediction of retina mechanics. Different impact scenarios were simulated using the FE method to obtain the von Mises stress map and the maximum principal strain map in the posterior retina. These stress and strain patterns, along with their input parameters, were used to train and test a partial least squares regression (PLSR) model to predict the soccer-induced retina stress and strain in terms of distributions and peak magnitudes. The peak von Mises stress and maximum principal strain prediction errors were 3.03% and 9.94% for the frontal impact and were 9.08% and 16.40% for the diagonal impact, respectively. The average prediction error of von Mises stress and the maximum principal strain were 15.62% and 21.15% for frontal impacts and were 10.77% and 21.78% for diagonal impacts, respectively. This work provides a surrogate model of FE analysis for the fast prediction of the dynamic mechanics of the retina in response to the soccer impact, which could be further utilized for developing a diagnostic tool for soccer-related ocular trauma.

## 1. Introduction

Soccer is the fastest growing youth sport, and soccer-related ocular injuries in young players have attracted increasing attention [1]. A fast-moving soccer ball hitting the eye could cause hyphemia, corneal abrasions, traumatic retinal edema, retinal hemorrhage, retinal detachment, macular hole, choroidal hemorrhages, and even impaired vision [2,3,4,5,6,7,8,9,10,11,12]. Horn et al. [11] reported thirteen soccer-related retinal injury cases, with half of these cases requiring surgical intervention. Filipe et al. [5] reported severe ocular injuries in soccer players at all skill levels, sometimes without early symptoms. Retinal hemorrhages on the posterior segment of the eye are a common ocular injury in youth soccer players [13]. It is critical to quantify the flying soccer ball-induced forces on the eyeball to better understand the mechanism of soccer-related ocular injuries. 

The finite element (FE) method has been a popular tool for quantifying the physics and mechanics of lesions and providing insights into injuries. Weaver et al. characterized the ocular lesion’s stresses, energy, and pressures associated with the risk of injury [14]. Stress and strain distributions were also obtained from FE models in abuse head trauma (AHT) [15,16,17,18,19,20]. Clemente et al. validated the FE simulation through in vitro experiments of bullet impacts on porcine eyeballs. They demonstrated that retinal injuries were caused by the tension resulting from the reflection of pressure waves [21]. Liu et al. performed an FE analysis of the impact of plastic pellets on the eyeball. They showed that the pressure wave propagation in the retina caused the retina to tear, and negative pressure contributed to the retina’s detachment [22]. Our group has also developed FE models to capture the pressure wave propagation in the vitreous and the impact on the retina to understand the mechanism of soccer-related retinal injuries [23]. These quantitative analyses have enhanced the mechanistic understanding of soccer-related retinal injuries. However, the computational cost hinders the direct application of FE analysis in clinical settings. 

The machine learning (ML) method, integrated with the FE method, could solve the problem of computational cost and instantly predict the FE results. The ML methods have been applied in biomedical research focusing on image processing and disease diagnosis, whereas the integration of FE and ML methods is still in its early stage. Gharaibeh et al. developed a deep learning approach for segmenting calcified coronary plaque from optical coherence tomography images [24]. ML has also been utilized to categorize glaucoma, diabetes, etc., based on fundus images [25,26]. FE data has been used to provide enriched datasets for ML models for predicting the Young’s Modulus and Poisson’s ratio of composite materials [27,28,29]. Our group also developed simulation-driven ML models for predicting the lumen area following the implantation of a medical device, stent. Our earlier work showed that the mechanistic understanding of the FE method could enhance the feature selection and prediction accuracy of ML models [30]. Liang et al. developed a deep learning approach to estimate the stress distribution in the thoracic aorta based on FE datasets [31]. This has demonstrated that the ML method could serve as a fast and reliable surrogate for the FE method. However, the application of this approach to retinal injuries is lacking.

In this work, we develop a surrogate model of FE analysis of soccer ball-induced retinal mechanics by combining the ML method with FE analysis. We construct a streamlined, three-dimensional eye model, including its essential components (i.e., the sclera, vitreous, retina, and retinal vessels), anatomically located in a rigid human skull, which is then subjected to impacts from a deformable soccer ball at six different velocities (30 mph, 32 mph, 34 mph, 38 mph, 50 mph, and 60 mph) along two different impact orientations (frontal and diagonal). FE analysis of 12 different impact scenarios are performed. The maximum principal strain and von Mises stress distributions in the posterior retina is extracted from the FE analysis as ML outputs. The FE input parameters (impact velocity and orientation) and outputs (distribution and values of von Mises stresses and maximum principal strains) were used to train the ML models and then predict/visualize the FE outputs in another three cases. 

## 2. Materials and Methods

**Figure 1:**
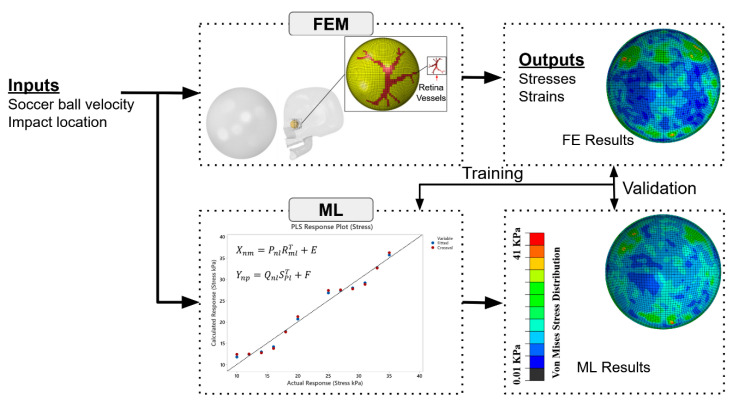
Overall framework to combine FE analysis with an ML method for rapidly predicting soccer-induced posterior retina stress/strain patterns.

The overall workflow of our framework, which integrates FE analysis with ML model for rapid prediction of soccer-related retinal injuries, is illustrated in Figure 1. There are two major steps: (1) FE analysis of soccer-related ocular injuries with streamlined models; (2) statistical model training with results from FE analysis for rapid prediction of ocular injury patterns.

### 2.1. FE Analysis

**Figure 2:**
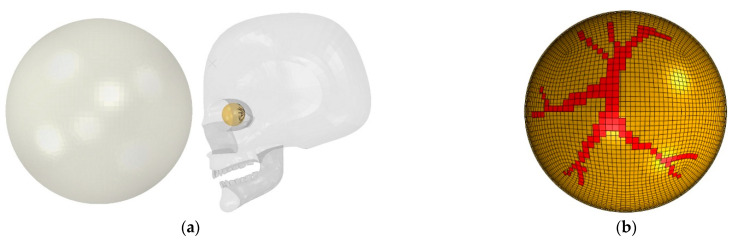
Finite element model of soccer ocular injuries. (**a**) Initial setup of a flying soccer ball hitting the face, including the eyeball; (**b**) retinal vessels in the posterior retina (frontal view).

A streamlined eye model of a young athlete was developed retaining the sclera, vitreous, retina, and retinal vessels, which was applied in our previous studies of retinal injuries [23,32,33]. The eyeball, located in the skull, was subjected to a deformable soccer ball (Figure 2a). The sclera and retina were simulated as hollow spheres with outer diameters of 26 mm and 24.5 mm, and thicknesses of 0.8 mm and 0.25 mm, respectively. The retina was filled with the vitreous [17,18,19]. Retina vessels were incorporated along the posterior retina and vitreous were highlighted in the posterior of the retina based on the standard fundus photograph [17,20], as shown in Figure 2b. A rigid human skull model was used from the library of Grab cad [34]. The eyeball position in the skull was 16 mm [35,36] between the corneal apex and the lateral orbital rim. A spherical shell with an outer diameter of 220 mm and a thickness of 0.8 mm [12] was considered as a standard soccer ball. We simulated both frontal and diagonal (45-degree angle) impacts. The soccer ball had an initial location of 15 mm in both frontal and diagonal to the apex of the eyeball and had six different velocities (30 mph, 32 mph, 34 mph, 38 mph, 50 mph, and 60 mph). The results from these 12 different impact scenarios were input into the statistical model training, as described in the next section.

Material properties of the eye model are summarized in Table 1. Sclera, vitreous, and retina were considered incompressible materials with a Poisson’s ratio of 0.49. The sclera was modeled as a hyperelastic material based on the published uniaxial test data [16]. Vitreous, composed of mostly water (>90%), was considered as a viscoelastic material, described by a Prony series expansion of the dimensionless relaxation modulus as:

(1) $$g_{R}\left(t\right)=1-\sum_{i=1}^{N}{\bar{g}}_{i}^{P}+\left(1-e^{-t/\tau_{i}^{G}}\right)\;$$

where material constants ${\bar{g}}_{i}^{\;P}\;$ and $\tau_{i}^{G}$ are 0.97 and 0.07, respectively. Retina was modeled as an elastic material with Young’s modulus of 20 KPa [37] and Poisson’s ratio of 0.49. The skull was considered a rigid body.

The surface-based fluid cavity technique was used to simulate the air pressurization of the soccer ball [38]. The pressure in the cavity of the soccer ball was filled with air. A cavity reference node with a single degree of freedom was chosen at the soccer ball’s center of gravity to represent the pressure inside the cavity [39].

Following the mesh convergence study, the number of elements for each component is shown in Table 1. The rigid skull was fixed [40]. Four points on the frontal plane of the sclera, which was 7.8 mm in front of the equator of the eyeball, were fixed to mimic the constraints of extraocular rectus muscles, which stabilize and control the movement of the eye in humans [17,20]. A penalty method with a friction coefficient of 0.3 was implemented among the soccer ball and eyeball. Frictionless contact was enforced between the retina and vitreous. Tie constraints were prescribed between the retinal vessel area and vitreous and between the retina and sclera. We used the final von Mises stress and maximum principal strain value at the end of the simulation in these 12 impact scenarios. The solutions were obtained using the dynamic explicit solver of a commercial Abaqus/Explicit software version 2019 (Dassault Systemes Simulia Corporation, Providence, RI, USA).

### 2.2. Partial Least Squares Regression (PLSR) Model Training

A PLSR model was trained using the data acquired from FE simulations of 12 different eye impact scenarios. The goal was to illustrate the effectiveness of our framework. The impact velocities and location were required inputs. Specifically, we used six different impact velocities from 30 mph to 60 mph and two different impact locations (frontal and diagonal).

PLSR is a multivariate technique that predicts response variables from predictor variables. The two fundamental equations in PLSR are the predictor matrix (*X*) and the response matrix (*Y*), given by [41]

(2) $$X_{nm}=P_{nl}R_{ml}^{T}+E$$

(3) $$Y_{np}=Q_{nl}S_{Pl}^{T}+F$$

where *P* and *Q* are the projection matrices, *R* and *S* are the transposed orthogonal loading matrices, where the rows are created from eigenvectors or principal components, and *E* and *F* are the error terms. X and Y are estimated using linear regression through

(4) $$Y=X\tilde{B\;}+{\tilde{B}}_{0}$$

where $\tilde{B\;}$ is the least squares regression estimate and ${\tilde{B\;}}_{0}\;$ is the prediction error. The input variables are the initial velocity and the impact location. The response variables are the von Mises stress and maximum principal strain within the elements of the posterior retina. The model prediction accuracy was tested by performing a ‘leave-one-out’ analysis, in which one simulation out of 12 was left out as the testing case of the PLSR model and then repeated for each scenario. The average errors between PLSR predictions and FE model results were estimated by comparing the von Mises stress values from PLSR and FE models at every element (total of 8322 elements) within the posterior retina. We used the PLSR plugins from the Python SciPy (www.scipy.org accessed on 1 December 2021) and scikit learn (machine learning) modules for the model training. We used python script in the Abaqus to extract stress and strain values from all the elements and automatically write the predicted stress and strain results in the Abaqus ODB file to compare visualization of the FE simulation results and predicted PLSR results. We used MySQL (www.mysql.org accessed on 1 December 2021) database connected to the PyCharm as an IDE for python programming to store all data, 

## 3. Results

**Figure 3:**
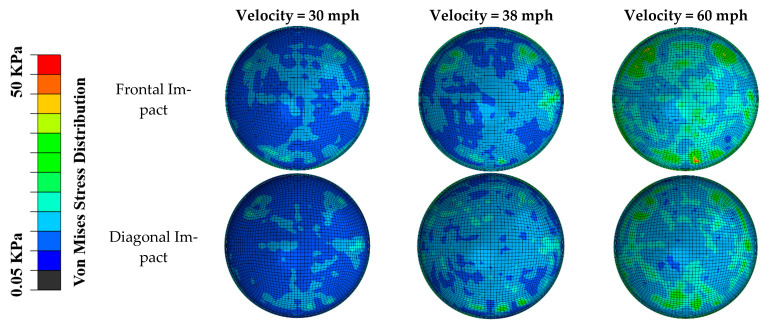
The distribution of von Mises stress in the posterior retina in frontal and diagonal impact scenarios at three different velocities.

**Figure 4:**
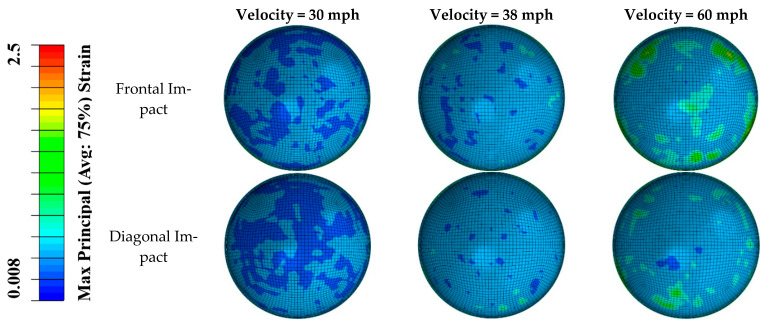
The distribution of maximum principal strain in the posterior retina in frontal and diagonal impact scenarios at three different velocities.

The distributions of von Mises stress and maximum principal strain in the posterior retina in different impact scenarios are shown in Figure 3 and Figure 4. Different stress/strain distribution patterns caused by frontal and diagonal impact indicate that the impact direction plays an essential role in the stress/strain distribution following impact. In both frontal and diagonal impact scenarios, as the soccer ball velocity increased to 60 mph, the maximum value of von Mises stress and maximum principal strain in the posterior retina reached 50 KPa and 2.5, respectively. The frontal impact caused higher stress and strain in the posterior retina than the diagonal impact did, especially at the retinal vessel bifurcation.

**Figure 5:**
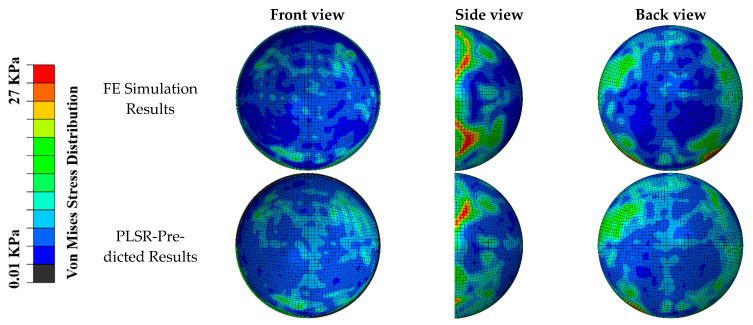
Comparison of FE simulated von Mises stress pattern and PLSR-predicted von Mises stress (soccer ball velocity was 35 mph with diagonal impact location). General agreements can be seen in the location and magnitude of maximum stress.

**Figure 6:**
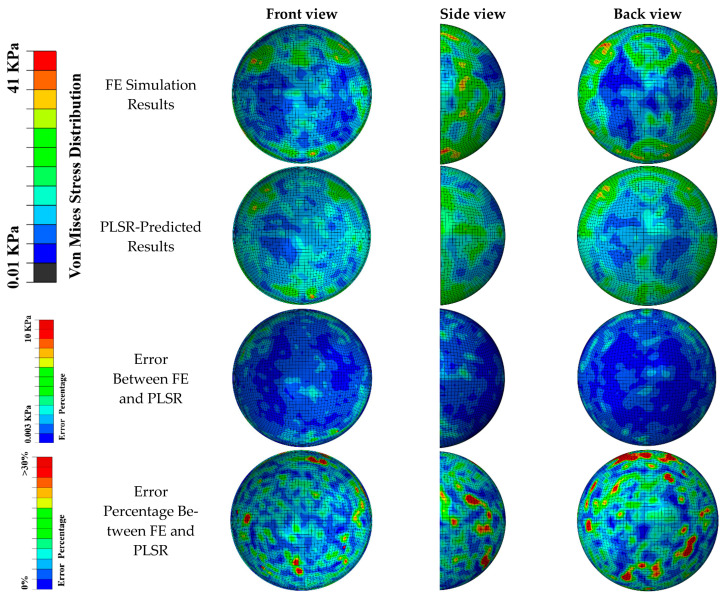
Comparison of FE simulated von Mises stress pattern and PLSR-predicted von Mises stress (soccer ball velocity was 65 mph and frontal impact location). General agreements can be seen in the location and magnitude of maximum stress.

**Figure 7:**
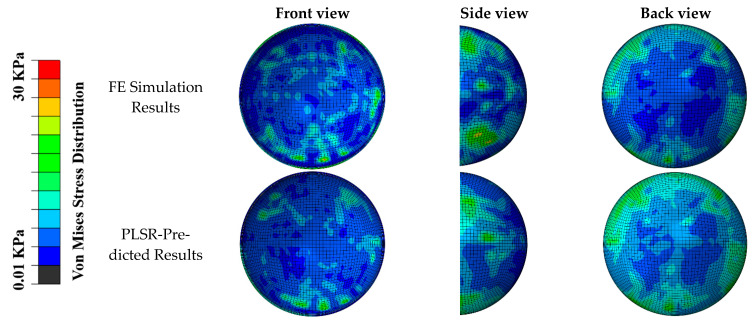
Comparison of FE simulated von Mises stress pattern, and PLSR-predicted von Mises stress (soccer ball velocity was 40 mph and 30-degree angle impact location). General agreements can be seen in the location and magnitude of maximum stress.

The distribution of the von Mises stress predicted by our PLSR-trained model was compared with the FE simulation (Figure 5 and Figure 6) in the scenarios of diagonal impact with a soccer ball velocity of 35 mph and frontal impact with a soccer ball velocity of 65 mph, respectively. Qualitative comparisons of the front, side, and back views showed that the PLSR-trained model captured the overall von Mises stress distribution pattern, including the locations of the greatest values. The prediction error and relative prediction error of the von Mises stress at each element in the retina are also shown in Figure 6. Only a small portion (less than 5%) of the elements had a relative prediction error larger than 30% due to the low-stress value from FE analysis (nearly 0 value). In addition, our PLSR prediction also captured the von Mises stress distribution for a 30-degree impact (Figure 7), an angle which was not included in the training dataset. This illustrates that our model can predict the stress with a new velocity and impact location which had never been seen before by our trained model. 

**Figure 8:**
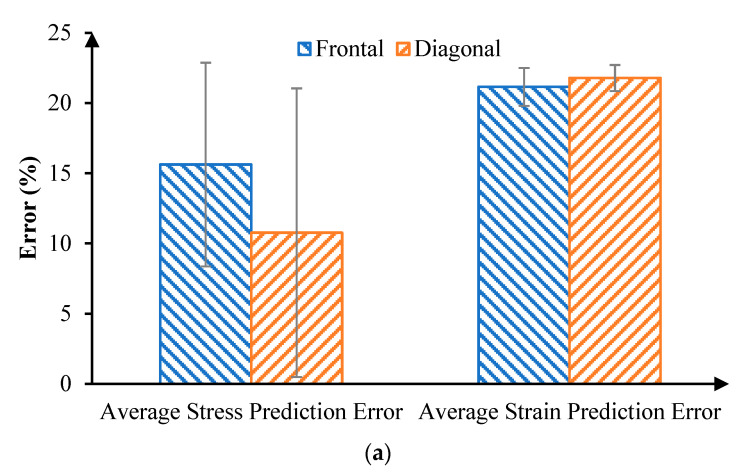

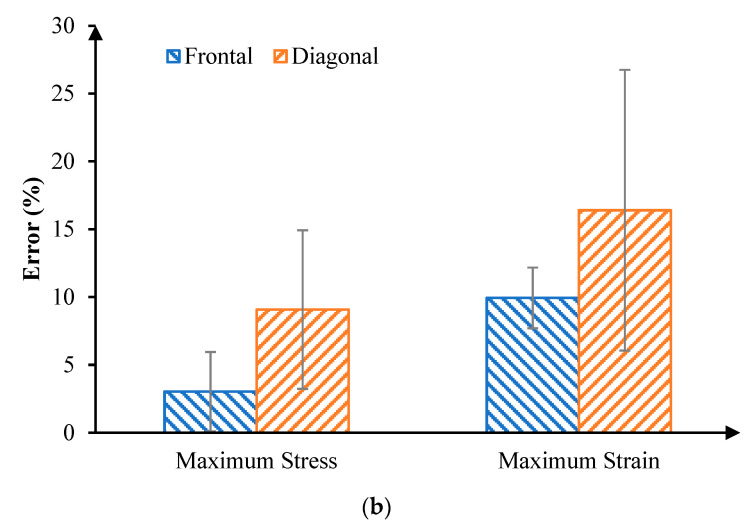
(**a**) Comparison of average stress and strain error between FE results and PLSR prediction results. (**b**) Comparison of maximum stress and strain predicted error.

The accuracy of our PLSR model predictions was evaluated by two different measures (Figure 8). First, we averaged the absolute difference of von Mises stress (or maximum principal strain) between FE and PLSR results in all elements, as the average error. Second, we compared the peak von Mises stress (or maximum principal strain) between FE and PLSR results for all cases, because the peak stress or strain have been associated with retinal injuries. The average error of von Mises stress between PLSR and FE models was 15.62% for frontal impact and 10.77% for diagonal impact. The average error of maximum principal strain between PLSR and FE models was 21.15% for frontal impact and 21.78% for diagonal impact. The error of peak von Mises stress were 3.03% and 9.08% for the frontal and diagonal impact, respectively. The error of peak maximum principal strain were 9.94% and 16.40% in frontal and diagonal impact, respectively. These results indicate that the stress and strain distributions, especially the peak stress and strain magnitudes, could be well predicted by our PLSR model.

## 4. Discussion

This study illustrated a framework that used an FE analysis of soccer ball impacts onto the eyeball to train ML models, which can serve as surrogate models for the rapid prediction of posterior retina mechanics following a soccer ball impact. Specifically, a PLSR model was trained and tested using the stress and strain datasets extracted from FE results. Then, we were able to very quickly estimate the stress and strain distributions, along with the peak stresses and strains, which is required for implementations in a clinical setting. The impact velocity and orientation were used to predict the retinal stress and strain distributions, including the location and magnitude of the peak von Mises stress and the maximum principal strain. The prediction error of the peak retinal von Mises stress was approximately 3.03% for frontal impact and 9.08% for diagonal impact. The corresponding prediction errors of the peak principal strain were 9.94% and 16.40%, respectively. To the best of our knowledge, this was the first study investigating the fast prediction of soccer ball-induced retinal injuries. The contribution of this work was the development of the combined FE and ML model to rapidly predict posterior retinal injuries following soccer ball impacts. 

Our FE results demonstrated that the von Mises stresses in the posterior retina were noticeably increased, especially with larger impact velocities and in regions interfaced with retina vessels and retina vessel bifurcations. In addition, the retinal stress and strain were aggregated by increasing the angle of impact from 45° (i.e., diagonal) to 0° (i.e., frontal). This agrees with observations by Karimi et al. [42]. We observed a peak von Mises stress of 50 kPa and an average von Mises stress over the posterior retina of 7.5 kPa for a soccer ball flying at 50 mph at an impact angle of 0°. Abnormally high von Mises stress and maximum principal strain have been linked with an elevated risk of ocular injuries [37,41]. Wollensak et al. [37] reported porcine retina fracture thresholds in terms of an engineering stress of 10 kPa and a strain of 42% based on 30 enucleated porcine eyes. We observed higher stress and strain values than the reported threshold values. The advantage of our approach lies in the fact that we extracted stress and strain values from all discretized elements of the posterior retina. This allowed us to utilize the stresses and strains of every element as training for the ML model, map all ML-predicted stresses and strains back to the posterior retina, and then examine the prediction errors in any element, individually or on average. 

To overcome the major drawback of FE analysis (i.e., the computational cost), we utilized sophisticated FE models of various soccer-related eye impact scenarios to train the ML-based models. We then used the ML models to rapidly predict the retinal von Mises stress and maximum principal strain distributions. Specifically, we adopted a PLSR method for training and prediction. The average accuracy for predicting the stress locations and magnitudes for all elements in the front and diagonal impact were 84.38% and 89.23%, respectively. The average accuracy for predicting the strain locations and magnitudes for all elements in the front and diagonal impact were 78.85% and 78.22%, respectively. Furthermore, the accuracies of the maximum stress and strain predictions were, respectively, 96.97% and 90.06% for frontal impact and 90.92% and 83.6% for diagonal impact. The reason that we obtained better accuracies in the predictions of stress than of strain is related to the deviation of the strain values (most of which were less than 0.1). There were higher fluctuations in the strain values than in the stress values, so we obtained better accuracy predicting stress than strain. This study provides a reasonable basis for developing an objective diagnostic tool for eye injuries that incorporates stress and strain markers for predicting retina damage.

Although our PLSR model predicted the stress and strain with satisfactory accuracy, the major limitation of this work is the relatively small number of FE simulations used for the training. The generalizability of our model could be enhanced with additional training datasets (i.e., FE simulations) that consider anatomical variations [43], such as different facial shapes and sizes, ages, genders, lesion properties, and impact orientations. A complicated eye model that considers the anterior chamber structures, such as the cornea, the iris, the lens, and the orbital fat (intraconal and extraconal), could mitigate the stress magnitude in the retina. The skull was simulated as a rigid body; a deformable skull might lead to smaller retinal stresses. Despite these simplifications, the present work demonstrated that a method integrating the FE and ML approaches can quickly predict posterior retina stress and strain patterns, which could be correlated with retinal hemorrhages and retinal detachment [23]. The fast prediction of stress and strain patterns with ML could also be expanded to other ocular components and improve the understanding of a variety of ocular injuries, such as those resulting from accidents or abusive head trauma. This work has the potential to assist clinicians in discerning the pathology of ocular injuries and promote a confident diagnosis.

## Figures and Tables

**Table 1:** Material parameters of the FE model and the number of elements.

Model Component	Element Type	Material Model	Number of Elements	$\mathrm{Density}\;(kg/m^{3})$	Material Parameters
Skull	R3D3	Rigid Body	18,103	-	-
Sclera	C3D8R	Hyperelastic	28,032	1243	[16]
Retina	C3D8R	Elastic	17,856 (8322 located at the posterior retina)	1000	E=20 KPa, ν=0.49 [37]
Vitreous	C3D8R	Viscoelastic	103,968	1009	E=43 Pa, ν=0.49$,\;{\bar{g}}_{i}^{\;P}$$=0.97,\;\tau_{i}^{G}$ = 0.07 [19]
Rubber soccer ball	S4R	Elastic shell (isotropy and homogeneity)	5001	1160	E=48 MPa, ν=0.49$,\;A=28.97\;g.{\mathrm{mol}}^{-1}$$,\;B=29.19\;g.{\mathrm{mol}}^{-1}$, C = 0.9 bar, T = 0.8 mm [38]

${\bar{g}}_{i}^{\;P}$ and $\tau_{i}^{G}$ = Prony series coefficients; A = ideal gas molecular weight; B = molar heat capacity; C = fluid cavity pressure; T = thickness, three-node; R3D3 = three-dimensional rigid triangular facet; C3D8R = an eight-node linear brick, reduced integration, hourglass control; S4R = a four-node doubly curved thin or thick shell, reduced integration, finite membrane strains.
